# IUC24358-87 Prognostic assessment of the Meet-URO score compared with the IMDC score in metastatic renal cell carcinoma (mRCC) receiving first-line systemic therapies (Meet-URO 33 study)

**DOI:** 10.1093/oncolo/oyaf248.043

**Published:** 2025-08-29

**Authors:** S E Rebuzzi, C Messina, L Bonomi, S Scagliarini, S Chiellino, B A Maiorano, F M Deppieri, A Cavo, V Conteduca, D Bimbatti

**Affiliations:** Medical Oncology Unit 2, Ospedale Molinette Azienda Ospedaliero-Universitaria Città della Salute e della Scienza di Torino, Torino, Italy; Oncology Unit, ARNAS Civico, Palermo, Italy; Unit of Oncology, ASST Papa Giovanni XXIII Hospital, Bergamo, Italy; UOC di Oncologia, Azienda Ospedaliera di Rilievo Nazionale Cardarelli, Napoli, Italy; Medical Oncology, I.R.C.C.S. San Matteo University Hospital Foundation, Pavia, Italy; IRCCS Ospedale San Raffaele, Milano, Italy; Ospedale dell’Angelo, AULSS 3 Serenissima, Mestre, Italy; Oncology Unit, Villa Scassi Hospital, Genova, Italy; Unit of Medical Oncology and Biomolecular Therapy, Department of Medical and Surgical Sciences, University of Foggia, Policlinico Riuniti, Foggia, Italy; Oncology Unit 1, Istituto Oncologico Veneto, IOV—IRCCS, Padova, Italy

## Abstract

**Background:**

The prognostic stratification is the cornerstone of treatment decision-making for mRCC. The novel Meet-URO score (IMDC score + NLR + Bone metastases) was developed in the immunotherapy era and has shown better prognostic performance compared with the IMDC score in different settings. Its application in the first-line IO-TKI setting was awaited.

**Methods:**

The Meet-URO 33 is a multicentric prospective observational study enrolling mRCC patients receiving first-line systemic therapy. A retrospective cohort of patients treated from 01.01.2021 was included. The Meet-URO score was assessed compared with the IMDC score in predicting OS. An exploratory analysis on PFS was also conducted.

**Results:**

A total of 1,557 patients were enrolled, 1400 (90%) were assessable. Median age was 66 years, 75% were males, 84% had clear cells, and 64% underwent nephrectomy; 20% received IO-IO, 66% IO-TKI (32% Pembrolizumab+Axitinib) and 14% TKI; 45% had NLR ≥ 3.2 and 29% bone metastases.

After a mFU of 14.1 months, mOS was 40.5 months, and mPFS was 16.8 months. The Meet-URO score confirmed a better prognostic stratification compared with the IMDC score (c-index 0.714 vs 0.688) (Table 1). Although the Meet-URO score was developed as an OS model, it showed a similar PFS performance (c-index 0.62 vs 0.61).

**Conclusions:**

The Meet-URO score confirmed its better prognostic accuracy compared with the IMDC score, also in a large-scale prospective cohort receiving first-line therapy. The adoption of the Meet-URO score should be implemented in clinical practice and as a stratification factor of clinical trials for more individualized patient management.

**Table:** 

**Score**	**Group distribution**	**HR (95%CI)**	***P* value**	**mOS (months)**	**3y-OS**
** Meet-URO **					
1	16%	Ref		NR	85%
2	30%	2.19 (1.34-3.56)	.002	NR	64%
3	22%	3.62 (2.22-5.90)	<.001	37.8	52%
4	24%	6.76 (4.25-10.75)	<.001	22.4	35%
5	8%	12.59 (7.54-21.04)	<.001	10.9	18%
** IMDC **					
Favorable	21%	Ref		NR	82%
Intermediate	57%	2.97 (1.99-4.42)	<.001	43.7	56%
Poor	22%	8.45 (5.61-12.70)	<.001	13.7	26%

